# Uncommon presentation of complicated internal hernia through the appendices epiploicae ring of adhesion: a clinical case study

**DOI:** 10.3389/fsurg.2023.1288369

**Published:** 2024-01-15

**Authors:** Mohamed Said Ghali, Mona S. Shehata, Mohammed Al Obahi, Raed M. Al-Zoubi, Ahmad Zarour

**Affiliations:** ^1^ Department of Surgery, Acute Care Surgery, Hamad Medical Corporation, Doha, Qatar; ^2^ Department of General Surgery, Ain Shams University, Cairo, Egypt; ^3^ Department of Pharmacy, Woman’s Wellness and Research Center, Hamad Medical Corporation, Doha, Qatar; ^4^ Surgical Research Section, Department of Surgery, Hamad Medical Corporation, Doha, Qatar; ^5^ Department of Biomedical Sciences, QU-Health, College of Health Sciences, Qatar University, Doha, Qatar; ^6^ Department of Surgery, Cornell Medical College, Doha, Qatar

**Keywords:** appendices epiploicae, surgery, adhesion internal hernia, falciform ligament, hernia

## Abstract

**Background:**

Internal hernias are infrequent, yet serious, medical conditions with potentially severe consequences. An internal hernia resulting from an Appendices epiploicae (AE) ring is an especially rare cause, with a mere nine documented instances worldwide.

**Case report:**

This report presents the case of a 58-year-old male who suffered from an internal hernia originating from an AE ring. The condition led to a gangrenous small bowel, which was treated by laparotomy and resection of the affected segment, followed by primary anastomosis. The post-operative recovery was favorable.

**Conclusion:**

Internal hernias, although rare, warrant immediate attention and early intervention to preclude detrimental outcomes, as illustrated in this particular case.

## Introduction

Internal hernias that result in small bowel obstruction (SBO) are exceedingly rare, particularly in an abdomen without prior surgical intervention (a “virgin abdomen”), as opposed to more frequent causes of SBO, such as adhesive SBO, abdominal wall hernias, or obstructive mass lesions (1). SBO accounts for 15% of global emergency surgeries, even though it ranks among the top reasons for hospital admissions by surgical units (2). Literature indicates the incidence of internal hernias at around 0.2%, with various causes including both congenital factors and post-surgical complications. An internal hernia can also occur through an Appendices epiploicae (AE) ring, following AE inflammation (appendagitis) which can cause one AE to stick to a neighboring one, or even without any previously known conditions (3). The rarity of AE ring-induced internal hernias has been highlighted in recent publications, showing that AE rings can form without prior episodes of appendagitis (3). The present report elaborates a case of intestinal obstruction with bowel gangrene due to an AE ring, with no history of abdominal surgery or symptoms resembling appendagitis in a virgin abdomen.

## Case report

A 58-year-old male patient, who had a medical history of diabetes mellitus, hypertension, and coronary artery disease, underwent coronary angiography and stenting 18 years earlier. He had not had any prior abdominal surgeries or trauma. The patient came to the emergency department with a 1-day history of abdominal pain, accompanied by vomiting and constipation, though he was able to pass a minimal amount of gas. There was no fever or other gastrointestinal symptoms. The pain was mainly located in the infraumbilical to suprapubic area, with a slight shift to the left iliac fossa, and was colicky in nature. A few hours after his arrival in the emergency room, he developed a rigid abdomen and was unable to move or cough. No additional system abnormalities were found.

**Figure 1:**
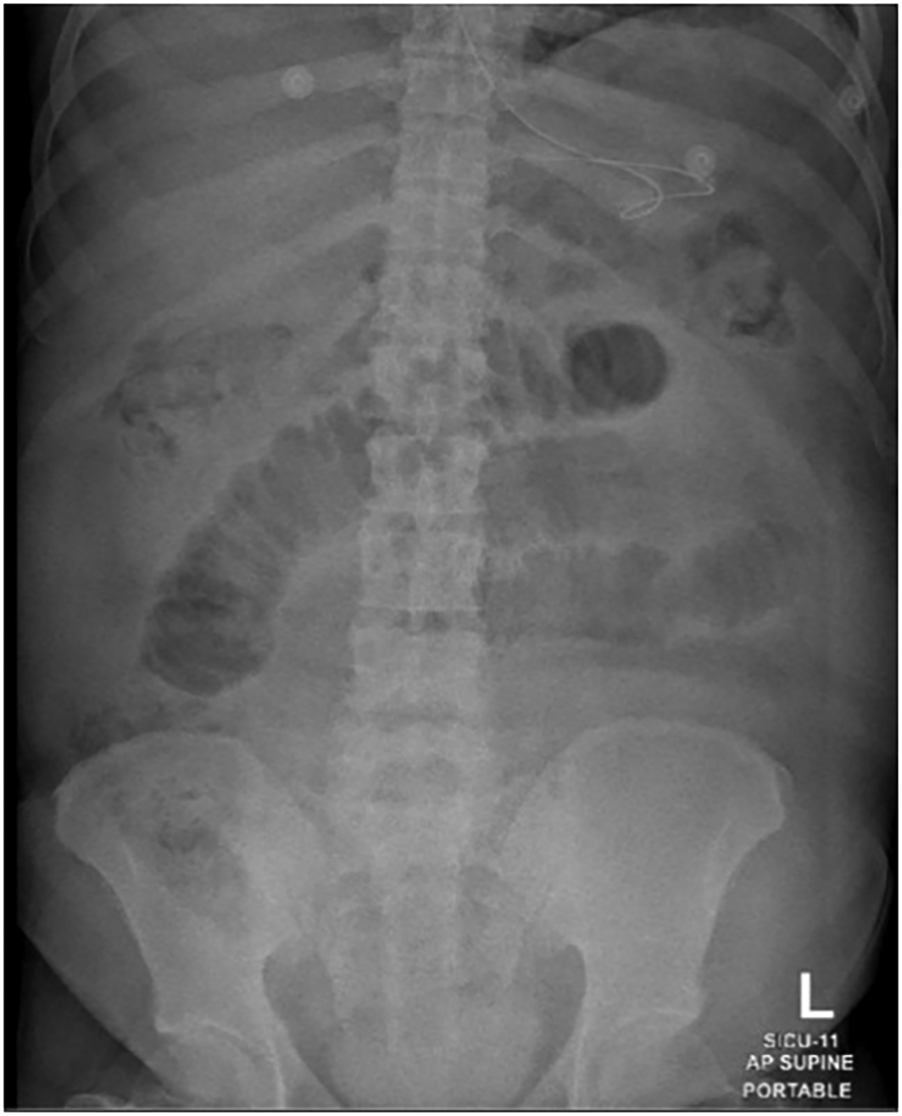
Demonstrating dilated jejunal small bowel loop.

His vital signs were normal. On examination, the patient's abdomen was distended, without scars, and displayed tenderness in the lower area, specifically towards the left iliac fossa. There was no rebound tenderness, but guarding was present in the abdominal muscles in this region. Percussion of the abdomen yielded a slight tympanic sound, and there was a decrease in bowel sounds. A digital rectal examination found normal-colored stool in a fully loaded rectum. Laboratory findings were mostly normal, except for an elevated white blood count of 13.2 × 10^3^ µl and slightly increased amylase and lipase levels at 179 and 149 µl, respectively. An abdominal plain x-ray showed dilated jejunal loops in the small intestine with a bowel diameter of 4.5 cm, mainly in the mid-abdomen, without air-fluid levels or free air (Figure 1).

**Figure 2:**
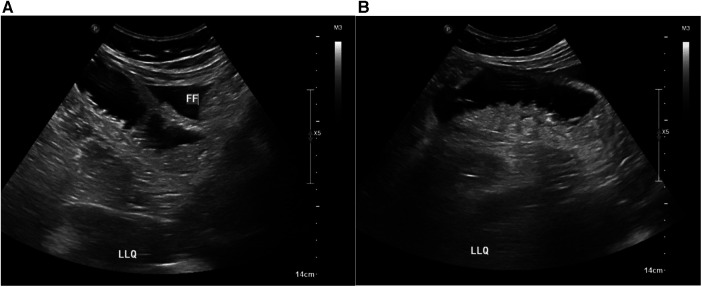
(**A**) Showing free fluid (FF) in the right iliac fossa, (**B**) dilated bowel loop.

While in the emergency department, the patient underwent an ultrasound, revealing mildly dilated, fluid-filled bowel loops and some free fluid in the left lower quadrant (Figure 2).

**Figure 3:**
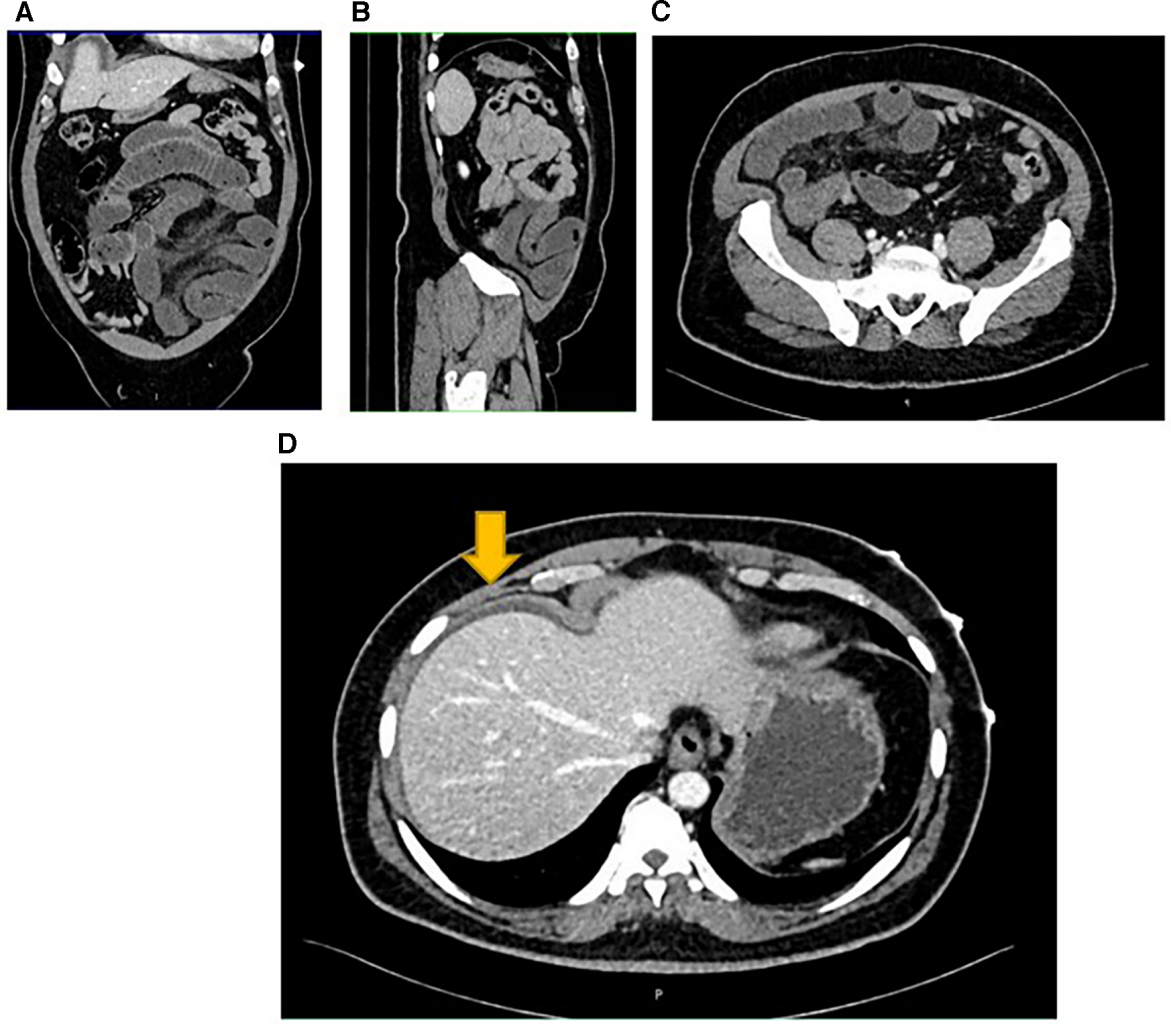
A transition point associated with dilated and collapsed small bowel loops (**A,B**). The Demonstrate mesenteric fat stranding (**C**) Perihepatic fluid demonstrated by the yellow arrow (**D**).

A CT scan of the abdomen with oral and intravenous contrast was performed to identify any surgical pathology. It revealed a transition point in the left mid-abdomen associated with dilated, fluid-filled proximal jejunal loops and collapsed distal small bowel loops. Also evident were long segment wall thickening of the distal jejunal/proximal ileal loop, mesenteric fat stranding, and a small amount of perihepatic fluid (Figure 3).

During the patient's examination, his vital signs started to show signs of decline, with a blood pressure reading of 80/60 mmHg, an elevated heart rate at 110 beats per minute, and increased lactate levels at 3 mmol/L. Consequently, he was transferred to the surgical critical care unit for urgent resuscitation. An abdominal examination revealed peritonitis and noticeable abdominal distension. Despite the offer of a nasogastric tube for abdominal decompression, the patient deteriorated. A comprehensive cardiology evaluation was performed, and the patient was prepared for an urgent exploratory laparotomy.

**Figure 4:**
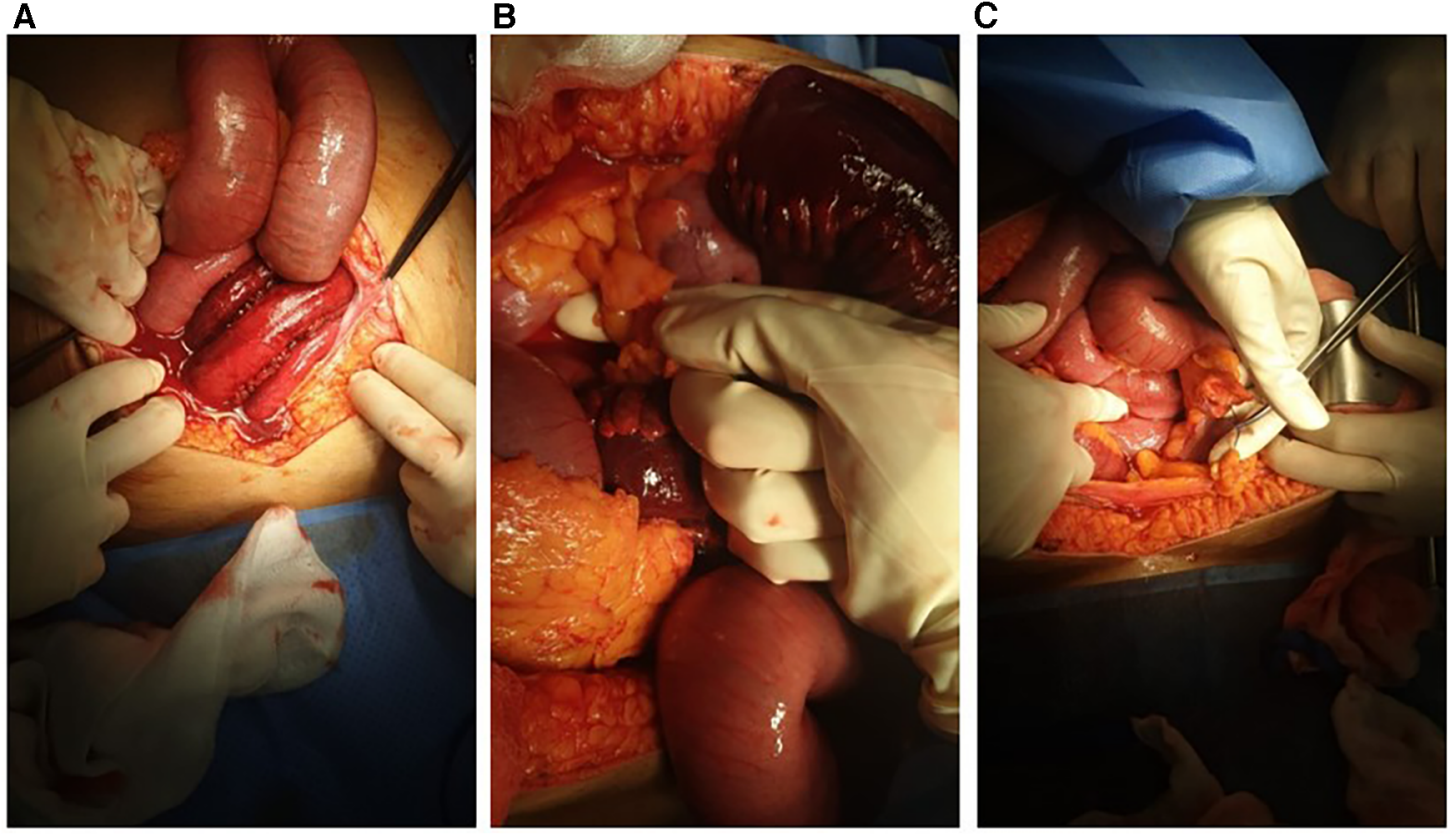
Hemorrhagic fluid and gangrenous bowel after opening the abdomen (**A**), appendices epiploicae ring before and after cutting it (**B,C**).

In the operation room, a midline exploratory laparotomy revealed a large volume of hemorrhagic fluid filling the entire abdominal cavity. The surgical findings also showed about 1 L of reactive hemorrhagic fluid and a notable adhesion between two Appendices epiploicae (AE) of the sigmoid colon. This adhesion had formed a ring that trapped and caused the small intestine to become gangrenous. Specifically, a 120 cm segment of the small bowel, located 50 cm from the ileocecal valve, was affected. The surgical team divided the AE ring to release the entrapped bowel, and the gangrenous portion of the bowel was removed and repaired with primary anastomosis (see Figure 4).

The patient was then transferred to the Intensive Care Unit (ICU) for management and correction of abnormal parameters. Following a satisfactory post-operative recovery, he was discharged in excellent health.

## Discussion

In this case, it is important to note that the appendices epiploicae are serosa-covered, fat-filled sacs protruding from the colon, each containing a central artery and vein. In an adult colon, there may be up to 100 AE, although their function is not yet understood (4). Appendagitis, or inflammation of the AE, can occur due to twisting or spontaneous venous thrombosis of the central vein. This condition is generally self-limiting and can often be managed conservatively. However, the symptoms can be misleading, which might lead to unnecessary hospitalizations and surgical interventions. Furthermore, appendagitis may result in adhesions between adjacent structures like the omentum or other AE, creating a ring through which the bowel may become entrapped, as evidenced in this case (5).

The AE ring can lead to intestinal obstruction, although there are documented instances where it has been discovered without preceding inflammation of the appendices epiploicae. Internal hernias are not common, and those caused by an AE ring are even rarer. Only a few of people have been diagnosed with this specific condition, typically after surgical exploration (2, 6).

In our particular case, we encountered this rare disorder without prior gastrointestinal (GI) complaints that might have indicated a history of appendagitis. Diagnosing this condition with radiology alone, based on symptoms of intermittent bowel obstruction, is challenging. Even in complex cases, imaging such as plain x-rays, ultrasound, and CT scans may only suggest small bowel obstruction without identifying the exact cause. In our patient, the scans revealed a dilated bowel, pointing to possible small bowel obstruction, but they did not provide definitive evidence of the underlying problem.

The AE ring's role as a cause of internal hernia was first identified in 1984 by Lau and Ong, and the eighth known case was published in 2022 by Zabihi et al. Our case has the distinction of being the first reported in the Middle East and North Africa in a native citizen rather than an immigrant (2, 7).

Treatment approaches for such cases can differ across various medical facilities. While laparoscopic exploration is the preferred approach at our institution, and other centers have managed similar cases in this way (8), we chose to perform a laparotomy right from the start. This decision was influenced by the patient's severe abdominal distension and critical condition before surgery.

## Conclusion

The case highlights the complexity of AE ring-induced internal hernia, underscored by the rarity of such instances and the challenges in diagnosis and treatment. The enigmatic nature of appendices epiploicae, their potential for inflammation-induced complications like intestinal obstruction, and the uniqueness of this case's presentation in the Middle East and North Africa emphasize the need for heightened clinical awareness and thorough diagnostics. The treatment variability across institutions underscores the absence of a standardized approach. Ultimately, this case contributes to our evolving medical knowledge, emphasizing interdisciplinary collaboration and ongoing research in unraveling the intricacies of rare anatomical anomalies and their clinical implications.

## Data Availability

The raw data supporting the conclusions of this article will be made available by the authors, without undue reservation.
